# Levetiracetam as a sensitizer of concurrent chemoradiotherapy in newly diagnosed glioblastoma: An open‐label phase 2 study

**DOI:** 10.1002/cam4.4454

**Published:** 2021-11-30

**Authors:** Kihwan Hwang, Junhyung Kim, Seok‐Gu Kang, Tae‐Young Jung, Jeong Hoon Kim, Se‐Hyuk Kim, Shin‐Hyuk Kang, Yong‐Kil Hong, Tae Min Kim, Yu Jung Kim, Byung Se Choi, Jong Hee Chang, Chae‐Yong Kim

**Affiliations:** ^1^ Department of Neurosurgery Internal Medicine Radiology Seoul National University Bundang Hospital Seoul National University College of Medicine Seongnam Republic of Korea; ^2^ Department of Neurosurgery Severance Hospital Yonsei University College of Medicine Seoul Republic of Korea; ^3^ Department of Neurosurgery Chonnam National University Hwasun Hospital Chonnam National University College of Medicine Hwasun Republic of Korea; ^4^ Department of Neurosurgery Asan Medical Center University of Ulsan College of Medicine Seoul Republic of Korea; ^5^ Department of Neurosurgery Ajou University Hospital Ajou University School of Medicine Suwon Republic of Korea; ^6^ Department of Neurosurgery Korea University Medical Center Korea University College of Medicine Seoul Republic of Korea; ^7^ Department of Neurosurgery Seoul St. Mary’s Hospital The Catholic University of Korea College of Medicine Seoul Republic of Korea; ^8^ Department of Internal Medicine Seoul National University Hospital Seoul National University College of Medicine Seoul Republic of Korea

**Keywords:** anti‐epileptic drug, concurrent chemoradiotherapy, drug repurposing, glioblastoma, levetiracetam

## Abstract

**Background:**

An open‐label single‐arm phase 2 study was conducted to evaluate the role of levetiracetam as a sensitizer of concurrent chemoradiotherapy (CCRT) for patients with newly diagnosed glioblastoma. This study aimed to determine the survival benefit of levetiracetam in conjunction with the standard treatment for glioblastoma.

**Methods:**

Major eligibility requirements included histologically proven glioblastoma in the supratentorial region, patients 18 years or older, and Eastern Cooperative Oncology Group (ECOG) performance status of 0–2. Levetiracetam was given at 1,000–2,000 mg daily in two divided doses during CCRT and adjuvant chemotherapy thereafter. The primary and the secondary endpoints were 6‐month progression‐free survival (6mo‐PFS) and 24‐month overall survival (24mo‐OS), respectively. Outcomes of the study group were compared to those of an external control group.

**Results:**

Between July 2016 and January 2019, 76 patients were enrolled, and 73 patients were included in the final analysis. The primary and secondary outcomes were improved in the study population compared to the external control (6mo‐PFS, 84.9% vs. 72.3%, *p* = 0.038; 24mo‐OS, 58.0% vs. 39.9%, *p* = 0.018), but the differences were less prominent in a propensity score‐matched analysis (6mo‐PFS, 88.0% vs. 76.9%, *p* = 0.071; 24mo‐OS, 57.1% vs. 38.8%, *p* = 0.054). In exploratory subgroup analyses, some results suggested that patients with ages under 65 years or unmethylated MGMT promoter might have a greater survival benefit from the use of levetiracetam.

**Conclusions:**

The use of levetiracetam during CCRT in patients with newly diagnosed glioblastoma may result in improved outcomes, but further investigations are warranted.

## Graphical abstract

Patients aged less than 65 years exhibited better outcomes with levetiracetam. Patients with unmethylated MGMT promoter possibly have greater benefits from levetiracetam.

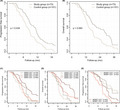

## 1 INTRODUCTION

Glioblastoma is an aggressive malignant brain tumor with a mortality rate that is the highest among all types of cancers in a human body.^1^ The current guideline for the standard treatment of glioblastoma, also known as Stupp protocol, has been shown to significantly improve the patient's outcome in the last decade^2^; however, glioblastoma often recurs within a year, and the life expectancy of the patients is less than two years in most cases.^3^ Several novel therapeutic approaches, such as patient‐specific tailored targeted therapy, immunotherapy, or blood‐brain barrier modulation, have been introduced for this refractory cancer, yet none of them are clinically applicable nor have been proven to improve survival.^4^

On the other hand, there has been high interest in existing non‐oncologic drugs for cancer management, referred to as repurposed drugs.^5^ For glioblastoma patients, it has been debated that the use of anti‐epileptic drugs (AEDs) in addition to conventional treatments would have a survival benefit.^6^, ^7^ Recently, most guidelines recommend against prescribing antiepileptic drugs with the intent either to prevent a seizure or to prolong survival.^8^, ^9^ Nevertheless, there is a lack of high‐level evidence to conclude, and it has still been an interesting issue if certain AEDs would have anti‐cancer effects. Since the recent generation of AEDs has limited adverse effects with adequate central nervous system penetration, numerous neuro‐oncologists have prescribed AEDs to glioblastoma patients without an indication for seizure control.

Levetiracetam, one of the most widely used AEDs in recent years, has been suggested to act as a sensitizer of concurrent chemoradiotherapy (CCRT) in glioblastomas. Therefore, we conducted a phase 2 study to validate the role of levetiracetam as a repurposed drug used in addition to the standard treatment of glioblastoma.

## 2 MATERIAL AND METHODS

### 2.1 Study design and eligibility criteria

This study is designed as a multicenter, single‐arm, open‐label, phase 2 study in Korea. Eight institutions have participated in this trial. The aim of the study is to validate the role of levetiracetam as a repurposed drug in the treatment of newly diagnosed glioblastoma.

Patients with histologically proven primary glioblastoma in the supratentorial region were eligible to participate in this study. Additional key inclusion criteria included age 18 years or older and Eastern Cooperative Oncology Group (ECOG) performance status of 0–2. The exclusion criteria consisted of any prior chemotherapy or radiotherapy, inadequate hematologic, renal, or hepatic function, unstable heart disease, serious neurologic or psychiatric disease, or an uncontrolled infection requiring treatment. Patients who had been exposed to the investigational agent within 30 days before enrollment were also excluded.

Each institution acquired approvals from its institutional review boards before enrollment started. All patients gave written informed consents according to national guidelines. Patients were not compensated for their participation.

### 2.2 Treatment

After the enrollment, the participants’ tumor samples from neurosurgical procedures were collected for assessment of molecular prognostic markers, including isocitrate dehydrogenase (IDH)‐1 mutation and *O^6^*‐*methylguanine*‐*DNA methyltransferase* promoter (MGMTp) methylation. The histopathologic diagnosis was established according to the 2007 and 2016 World Health Organization classifications.

All patients received the temozolomide‐based standard treatment of glioblastoma following tumor resection. For concurrent chemoradiotherapy (CCRT), patients received fractionated focal irradiation in daily fractions of 2 Gy for 6 weeks, for a total of 60 Gy. Concurrent temozolomide was given daily from the first to the last day of radiotherapy, at a dose of 75 mg/m^2^ body surface area. Adjuvant temozolomide chemotherapy was started 4 weeks after completion of radiotherapy and conducted for a maximum of 6 cycles. Patients received 150 mg/m^2^ of temozolomide for five days of the first cycle and 200 mg/m^2^ on the same schedule in subsequent cycles.

Levetiracetam was started with 250 mg twice a day and increased up to 1,000 mg/day during the perioperative period. For a couple of immediate postoperative days, the drug was administered intravenously mixed with 100–150 ml of normal saline. The drug was administered by oral tablets or syrup during CCRT, depending on the patient's condition. Participants who experience any events of clinical seizure received an increased dose of up to 2,000 mg/day and other additional AEDs under each clinician's decision. During adjuvant chemotherapy with temozolomide, a minimum daily dose of 1,000 mg was administered, unless there were serious side effects related to levetiracetam.

### 2.3 Outcome assessment

The primary endpoint was progression‐free survival (PFS) at 6 months (6mo‐PFS). PFS was calculated from the date of pathologic confirmation by operation. The second endpoint was overall survival (OS) at 24 months (24mo‐OS). OS was defined as the date of operation to the date of death from any cause. Landmark PFS at 6, 12, 18, and 24 months and OS at 12 and 24 months were evaluated. Patients were followed up weekly during postoperative CCRT, biweekly during adjuvant chemotherapy, and every 3 months after completion of the standard treatment if the disease was stable.

Magnetic resonance imaging (MRI) scans were performed 4 weeks after the end of CCRT prior to the first adjuvant treatment cycle, every 3 months during the first year thereafter, and every 4 months during the second year. Treatment response was evaluated using the criteria proposed by the Response Assessment in Neuro‐Oncology working group.^10^ Patients with possible pseudoprogression were kept on current treatment and re‐evaluated 4 weeks thereafter to clarify the response.

### 2.4 Statistical consideration

Datasets for the control group were collected from the institutional temozolomide chemotherapy registry. Baseline characteristics of the study group were compared to the external control group using Pearson's chi‐squared test for categorical variables and Wilcoxon's rank‐sum test for continuous variables. Cumulative survivals were estimated using the Kaplan–Meier method and differences were assessed with the stratified log‐rank test. Cumulative survival rates at index time points (6, 12, 18, and 24 months for PFS and 12 and 24 months for OS) were compared using the two‐proportion z test.

To validate the benefit of the interventional drug, outcomes were stratified by baseline variables. Continuous or categorical variables were dichotomized by widely accepted reference values in most clinical trials based on the Radiation Therapy Oncology Group recursive partitioning analysis classification for glioblastoma^11^ or a recent multicenter study with a similar patient population in Korea.^3^ Imbalances of baseline variables between the study and the external control populations were minimized by full optimal matching on the propensity scores.^12^ By this algorithm, all individuals were clustered by similar propensity scores and assigned a weight between 0 and 1 so that all clusters were balanced. The propensity scores were calculated by logistic regression to estimate the probability of the assignment to the study group on a basis of the following baseline variables: age at diagnosis (<65 or ≥65 years), gender, ECOG performance status (<1 or ≥1), and extent of resection (gross total resection, subtotal or partial resection, or biopsy only). The molecular prognostic factors, IDH and MGMTp statuses, were not matched due to the limited data availability of the external datasets. A standardized mean difference of 0.2 or less was considered as a negligible imbalance. After matching, the weighted Cox proportional hazard models were applied with robust variance estimates. The covariates were included in the multivariate models for subgroup analysis if they were significant in the full model.

Statistical analyses were performed using R v4.0.4 (R Foundation for Statistical Computing, Vienna, Austria). Each result from statistical tests was considered statistically significant if a two‐tailed *p*‐value was <0.05.

## 3 RESULTS

### 3.1 Study group

**FIGURE 1:**
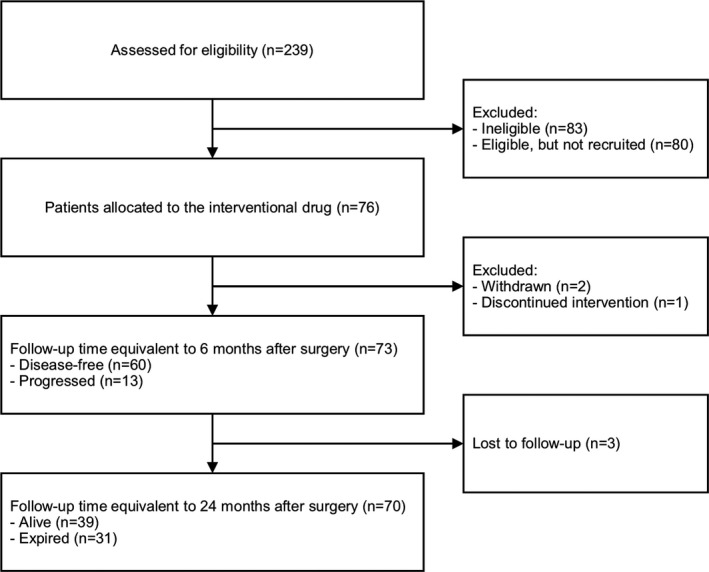
Flow diagram for Consolidated Standards of Reporting Trials (CONSORT) statement

Between July 2016 and January 2019, 76 patients who met the eligibility requirements consented to participate (Figure 1). Before the primary endpoint, two patients were dropped from the study: one patient had withdrawn and one patient who had developed acute kidney injury during CCRT had to discontinue the interventional drug. Before the secondary endpoint, one patient had further withdrawn. Overall, 73 patients were included in the final analysis (Table 1).

**TABLE 1:** Baseline characteristics

Variables	Study population (*n* = 73)
Age at diagnosis (year)	56.0 (46.0–64.0)
≥65	16 (22)
Gender	
Male	47 (64)
ECOG performance status	
0	16 (22)
1	49 (67)
2	8 (11)
Extent of resection	
Gross total resection	50 (69)
Subtotal or partial resection	18 (25)
Biopsy only	5 (7)
IDH status	
IDH‐wildtype	70 (96)
IDH‐mutant	3 (4)
MGMTp status	
Unmethylated	43 (59)
Methylated	27 (37)
Missing	3 (4)

Note

Values are medians (range) or numbers (%).

Abbreviations: ECOG, Eastern Cooperative Oncology Group; IDH, isocitrate dehydrogenase; MGMTp, *O^6^*‐*methylguanine*‐*DNA methyltransferase* promotor.

All patients completed CCRT and 54 (74.0%) patients completed six cycles of temozolomide adjuvant chemotherapy. All patients have maintained the interventional drug regardless of postoperative seizures. Six (8.2%) patients received 2,000 mg/day of levetiracetam, whereas the majority received 1,000 mg/day in two divided doses. All patients had progression of their disease despite the treatment and 43 (58.9%) expired during follow‐up. No patients reported serious side effects or overdose symptoms of levetiracetam, including fever, rash, mental alteration, agitation, or other behavioral changes. The median follow‐up of the study group was 26.5 (range, 15.0–32.0) months.

### 3.2 Control group

Clinical information in the external dataset from the temozolomide chemotherapy registry was retrospectively reviewed (Figure S1). All patients with newly diagnosed glioblastoma in this registry received the same standard postoperative treatment with Stupp regimen as described above, between June 2004 and May 2019. Of 261 individuals, a total of 26 samples were excluded due to the following reasons: prior chemotherapy or radiotherapy (*n* = 7), declined to receive the standard treatment during CCRT (*n* = 2), treated without concurrent temozolomide due to medical condition (*n* = 13), and treated with other adjuvant chemotherapies rather than temozolomide (*n* = 4). We screened the remaining 235 samples and identified 101 individuals with available clinical information who had never used levetiracetam during CCRT. Patients who were lost to follow‐up after hopeless discharge in a terminal stage were assumed to have expired on the date of the latest contact.

Baseline characteristics in the external control were compared to the study population (Table S1). Particularly, the extent of resection, a known major prognostic factor, was unequally distributed between two groups (*p* < 0.001), which might be required to further stratified and matched analysis to eliminate the selection bias. All individuals in the control group completed CCRT and 42 (41.6%) patients completed six cycles of temozolomide adjuvant chemotherapy. Sixty‐nine (68.3%) patients received valproic acid with a dose range of 900–1500 mg/day, three of which used multiple AEDs. Eight (7.9%) patients received topiramate, lacosamide, or others, whereas no AEDs were administered in 24 (23.7%) patients. During a median follow‐up of 18.4 (11.9–30.3) months, 89 (88.1%) patients had a recurrence and 85 (88.1%) patients expired.

### 3.3 Survival outcome

**FIGURE 2:**
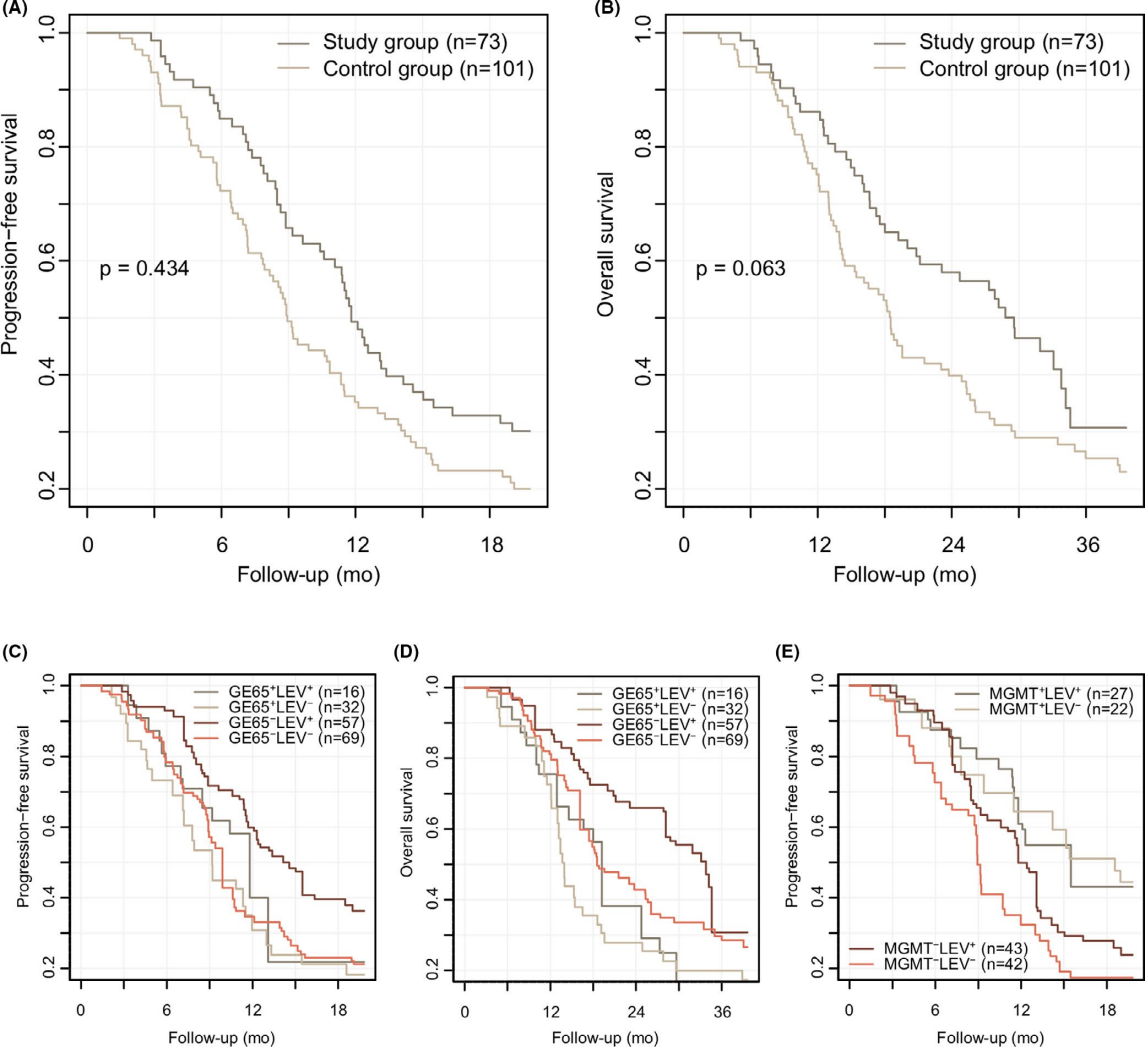
Kaplan–Meier curves for progression‐free and overall survival. The study group exhibited a slightly improved survival compared to the control group at primary (6mo‐PFS) and secondary (24mo‐OS) endpoints (A, B). Differences between the study and the control groups were still prominent in the younger population aged less than 65 years (C, D). Survival curves also displayed differential survival outcomes according to MGMTp status (E). Interestingly, individuals with unmethylated MGMTp who received levetiracetam (MGMT^−^LEV⁺) showed slightly improved PFS compared to those counterparts from the control group (MGMT^−^LEV^−^). GE65⁺, aged ≥65 years; GE65^−^, aged <65 years; MGMT⁺, methylated; MGMT^−^, unmethylated; LEV⁺, study group (with levetiracetam), LEV^−^, control group (without levetiracetam)

The Kaplan–Meier estimate of PFS for the study group was median 11.8 (95% CI, 11.1–14.6) months, which was slightly higher than the control group (Figure 2A), 8.9 (7.9–11.3) months, or the previous report,^3^ 10.1 (9.3–10.9) months. The Kaplan–Meier estimate of OS for the study group was 29.6 (21.1–34.6) months, which was also prolonged compared to both the control groups (Figure 2B), 18.5 (15.3–24.9) months, or the other study,^3^ 17.5 (16.5–18.5) months. Most glioblastoma patients had progressed and expired during follow‐up eventually, and the log‐rank test did not show any significant difference between the study and the control groups, accordingly.

Cumulative survival estimates at index time points were compared between two groups (Table 2). The primary and secondary outcomes were improved in the study population compared to the external control: 6mo‐PFS, 84.9% vs. 72.3% (*p* = 0.038); 24mo‐OS, 58.0% vs. 39.9% (*p* = 0.018). Furthermore, the outcome in this study was outperformed compared to the previous result from a multicenter study for glioblastoma: 24mo‐OS, 36.0%.^3^ However, differences between two groups were less prominent in a propensity score‐matched analysis, which is described in subgroup analysis below (Table 3): 6mo‐PFS, 88.0% vs. 76.9% (*p* = 0.071); 24mo‐OS, 57.1% vs. 38.8% (*p* = 0.054).

**TABLE 2:** Kaplan–Meier survival estimates at index time points

	Study population	External control	*p*‐value
PFS (%)			
At 6 months	84.9 [77.1, 93.5]	72.3 [64.1, 81.6]	0.038
At 12 months	49.3 [39.1, 62.2]	35.3 [27.0, 46.0]	0.063
At 18 months	32.9 [23.7, 45.6]	23.2 [16.2, 33.1]	0.161
At 24 months	20.5 [13.1, 32.3]	15.3 [9.5, 24.5]	0.378
OS (%)			
At 12 months	86.1 [78.5, 94.5]	75.1 [67.1, 84.1]	0.064
At 24 months	58.0 [47.5, 70.6]	39.9 [31.3, 50.8]	0.018

Note

Data are shown as numbers [95% CI].

Abbreviations: OS, overall survival; PFS, progression‐free survival.

**TABLE 3:**
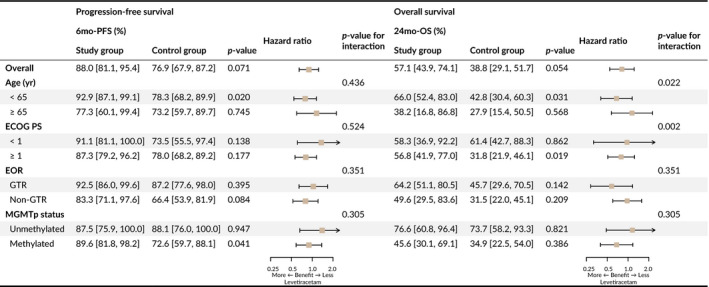
Subgroup analysis of progression‐free and overall survivals according to the use of levetiracetam

### 3.4 Subgroup analysis

As an exploratory analysis, survival outcomes of the study and the control groups were compared within each subgroup stratified by known prognostic factors. Prior to the analysis, all samples were weighed by full matching on the propensity score (Table S1), according to the method previously described. In the original model, extent of resection contributed as a major confounder in multivariate Cox regression: hazard ratios for PFS, biopsy (reference: gross total resection), 2.42 (95% CI, 1.43–4.10, *p* < 0.001), subtotal or partial resection, 1.47 (1.02–2.12, *p* = 0.038); hazard ratios for OS, biopsy, 3.14 (1.83–5.37, *p* < 0.001), subtotal or partial resection, 1.71 (1.13–2.59, *p* = 0.012). Other significant factors were as follows: aged ≥65 years (reference: <65), hazard ratio for OS, 1.93 (1.30–2.87, *p* = 0.001); male as gender (reference: female), PFS, 1.46 (1.05–2.03, *p* = 0.026), OS, 2.18 (1.48–3.22, *p* < 0.001); ECOG performance status of ≥1 (reference: <1), OS, 1.57 (1.01–2.44, *p* = 0.044).

Cox proportional hazard models of PFS and OS were implemented to validate the benefit of levetiracetam according to each subgroup, together with Kaplan–Meier estimates for the primary and secondary outcomes (Table 3). The benefit of levetiracetam was more prominent in the subgroup aged <65 year (Figure 2C–D): 6mo‐PFS, 92.9% vs. 78.3% (*p* = 0.020); 24mo‐OS, 66.0% vs. 42.8% (*p* = 0.031). Interestingly, the subgroup with unmethylated MGMTp also exhibited a significantly improved survival prognosis according to the use of levetiracetam (Figure 2E): 6mo‐PFS, 89.6% vs. 72.6% (*p* = 0.041). Other results were inconsistent to show a clear benefit from the use of levetiracetam.

## 4 DISCUSSION

Our analyses described how differences were exhibited according to several treatment factors, particularly focused on the use of levetiracetam. One of the limitations in this study includes the point that the external and the historical controls were implemented for comparative analysis with the study group. However, regarding the common beliefs of the patients and the clinicians’ judgments who experienced recent improvement of survival on glioblastoma patients in the same era of the newer generation of AEDs, a double‐blinded randomized clinical trial with placebos could not be easily conducted in glioblastoma patients, since an unfavorable prognosis is typically expected. Indeed, this study is limited by a single‐arm study design and a relatively small sample size. Despite the small sample size of this study, we observed almost generalized baseline characteristics of glioblastoma in the study population and the external control dataset after proper stratification and matching strategy.

In our study, the effect of levetiracetam on survival was minimal, and the difference between the study and the control groups was not prominent. On the other hand, known prognostic factors, specifically the extent of resection, behaved as a major confounder in both PFS and OS. There is no doubt that the role of a repurposed drug cannot exceed the importance of surgical resection in glioblastoma treatment. Nevertheless, there was a mild tendency to prolong the patient's survival even after correction of operation‐related confounding factors, which at least informs a few possibilities of an additive role of the interventional drug in the conventional treatment in a certain subpopulation, specifically the patients under the age of 65 years or with unmethylated MGMTp.

Several previous studies have demonstrated that levetiracetam helps patients with high‐grade gliomas, more than its anti‐epileptic activity. It has been suggested that levetiracetam enhances p53‐mediated inhibition of MGMT expression and therefore sensitizes the efficacy of temozolomide.^13^ Some recent *in vitro* experiments also demonstrated that levetiracetam enhances the effect of temozolomide or other anticancer drugs.^14^, ^15^ In our previous papers, levetiracetam exhibited a significant benefit in glioblastoma patients based on retrospective studies in each institution.^16^, ^17^ One study reported that levetiracetam presented a survival benefit in glioblastoma patients with methylated MGMTp,^18^ which is opposite to our observations in this study. Nevertheless, our results of differential outcomes according to MGMTp status may better enhance the hypothesis of levetiracetam acting as a sensitizer of CCRT by inhibiting MGMT expression through a different mechanism other than methylation.

There are other AEDs that have been described to have a potential anti‐cancer effect. For example, perampanel has been suggested to have a synergistic effect with temozolomide in vitro experiments,^19^, ^20^ although it failed to attenuate tumor growth in vivo models.^21^ Valproic acid has been observed to increase the survival of glioblastoma patients in several reports.^22^, ^23^ The effect of valproic acid on survival could be explained by its potential characteristic as a histone deacetylase inhibitor leading to epigenetic modifications,^24^ which might affect MGMTp status in glioblastoma.^25^ To the best of our knowledge, however, their differential activities according to MGMTp status have not been investigated yet.

A large study based on a pooled analysis of multiple clinical trials in glioblastoma revealed that neither levetiracetam nor valproic acid was associated with improved survival.^7^ On the contrary, a recent meta‐analysis concluded that levetiracetam might improve the prognosis of glioblastoma patients.^26^ The results from other clinical trials should be taken with caution in terms of the reliability of the data since those trials did not provide any detailed information on how each antiepileptic drug was administered during concurrent chemoradiotherapy or adjuvant chemotherapy. The AED as a repurposed drug might demonstrate a minimal improvement that cannot be easily observed during rapid clinical progress in glioblastoma patients. Another issue is that there has been a lack of evidence on the optimal dosage and dosing period of levetiracetam for survival benefits. Our study is also limited by the adoption of the common dose range for seizure control, which should be investigated in further studies. To summarize, the anti‐cancer effects of antiepileptic drugs are inconclusive based on the current evidence.

In a different context, there has been a positive point of view in using AEDs, especially the newer generation of AEDs, such as levetiracetam, that has shown an acceptable safety profile.^27^ In addition, it has been documented that levetiracetam improves cognitive function in high‐grade glioma patients^28^ and is a practical choice of AEDs in elderly patients with cognitive impairment.^29^ Since physiologic and cellular mechanisms of AEDs in the central nervous system are not fully understood, the role of AEDs on disease control and survival should be validated by further clinical and scientific investigations.

Given the circumstances, levetiracetam could be a reasonable option for patients with newly diagnosed glioblastoma where AED needs to be started, depending on the clinician's preference. Nevertheless, levetiracetam as a repurposed drug lacks strong evidence to recommend at this time, which additional research designed with a specific age or molecular target and a randomized control is warranted.

## 5 CONCLUSION

Levetiracetam might have a helpful role in the treatment of glioblastoma, which merits further investigations.

## CONFLICT OF INTEREST

The authors have no conflicts of interest to declare.

## AUTHOR CONTRIBUTION

Conception and design: Jong Hee Chang and Chae‐Yong Kim. Acquisition of data: all authors. Analysis and interpretation of data: Kihwan Hwang and Junhyung Kim. Drafting and revision of the manuscript: all authors. Final approval: all authors.

## ETHICS APPROVAL

This study was approved by the ethics committee of Seoul National University Bundang Hospital (B‐1601/329‐003).

## Supporting information

Supplementary Material

## Data Availability

Data are available on request due to ethical restrictions.
