# Supplementary material for: Levetiracetam as a sensitizer of concurrent chemoradiotherapy in newly diagnosed glioblastoma: An open‐label phase 2 study
Source: Cancer Med. 2021 Nov 30;11(2):371–9. doi: 10.1002/cam4.4454 (PMC8729048; doi:10.1002/cam4.4454)
Supplement: Supplementary file 1 — Supplementary Material [file CAM4-11-371-s001.docx]

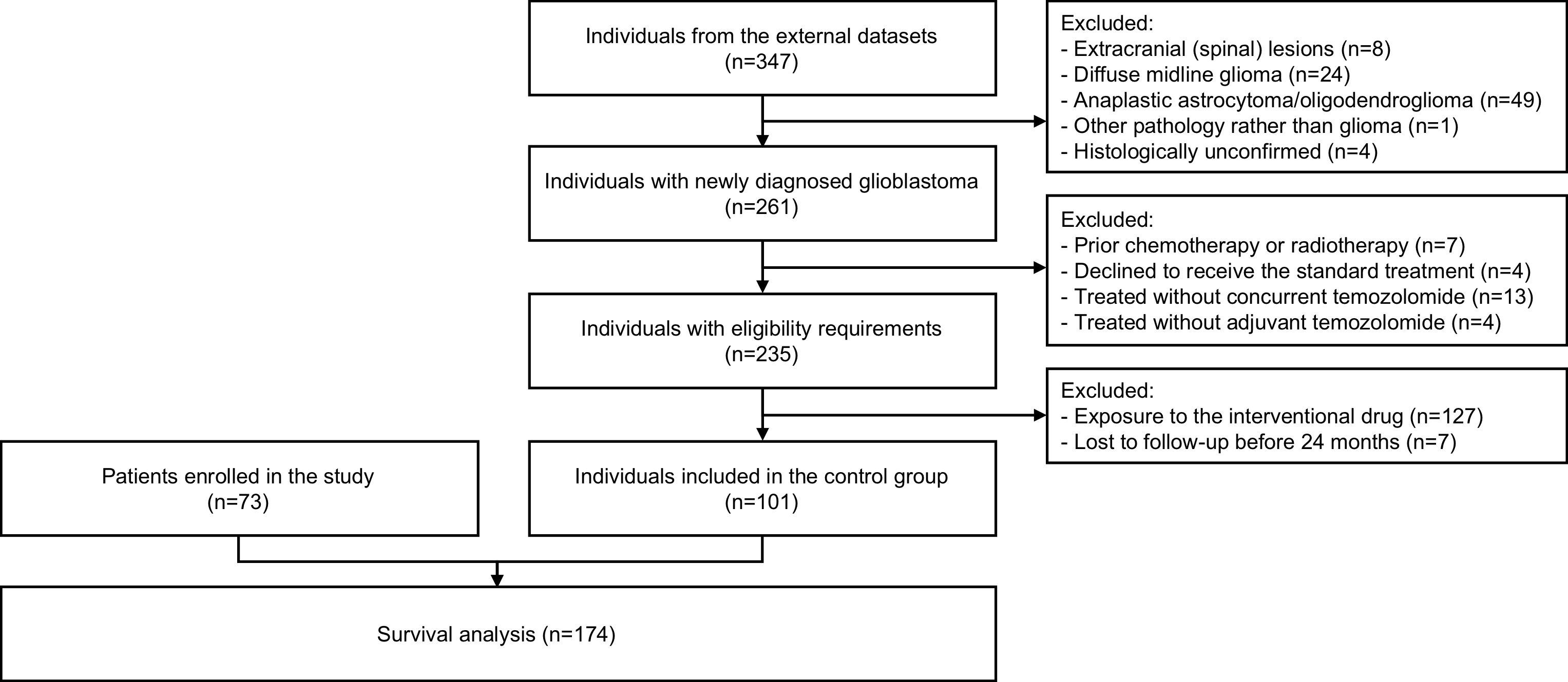


Figure S1. Selection of the external control group.

Table S1. Baseline variables and survival outcomes of the study and the control groups after full optimal matching on the propensity scores.

| **Variables** | **Study group (*n*=73)** | **Control group (*n*=101)** | **SMD** | |
| --- | --- | --- | --- | --- |
|  |  |  | **before matching** | **after matching** |
| **Age at diagnosis (year)** | 56.0 (46.0-64.0) | 60.0 (50.0-66.0) |  |  |
| ≥ 65 | 16 (21.9) | 32 (31.7) | -0.222 | 0.106 |
| **Gender** |  |  |  |  |
| Male | 47 (64.4) | 52 (51.5) | 0.264 | -0.066 |
| **ECOG performance status** |  |  |  |  |
| ≥ 1 | 57 (78.1) | 78 (77.2) | 0.021 | 0.117 |
| **Extent of resection** |  |  |  |  |
| Gross total resection | 50 (68.5) | 38 (37.6) | 0.650 | 0.000 |
| Subtotal or partial resection | 18 (24.7) | 39 (38.6) | -0.304 | 0.056 |
| Biopsy only | 5 (6.8) | 24 (23.8) | -0.483 | -0.073 |
| **IDH status** |  |  |  |  |
| IDH-wildtype | 70 (95.9) | 49 (48.5) | . | . |
| IDH-mutant | 3 (4.1) | 1 (1.0) | . | . |
| NOS | 0 (0.0) | 51 (50.5) | . | . |
| **MGMTp status** |  |  |  |  |
| Unmethylated | 43 (58.9) | 42 (41.6) | . | . |
| Methylated | 27 (37.0) | 22 (21.8) | . | . |
| Missing | 3 (4.1) | 37 (36.6) | . | . |
| **Outcomes** | **Study group** | **Control group** | **p-value** | |
| **Progression-free survival** | | | | |
| At 6 mo (%) | 88.0 [81.1, 95.4] | 76.9 [67.9, 87.2] | 0.071 | |
| At 12 mo (%) | 53.6 [41.2, 69.8] | 33.7 [24.8, 45.6] | 0.025 | |
| At 18 mo (%) | 34.0 [22.6, 51.1] | 22.6 [15.5, 33.0] | 0.170 | |
| At 24 mo (%) | 16.6 [09.6, 28.6] | 15.9 [09.5, 26.6] | 0.912 | |
| **Overall survival** | | | | |
| At 12 mo (%) | 84.0 [73.6, 95.9] | 78.9 [71.1, 87.5] | 0.462 | |
| At 24 mo (%) | 57.1 [43.9, 74.1] | 38.8 [29.1, 51.7] | 0.054 | |

Data are shown as median (range), number (%), or number [95% CI].

Abbreviations: ECOG, Eastern Cooperative Oncology Group; IDH, isocitrate dehydrogenase; MGMTp, *O^6^-methylguanine-DNA methyltransferase* promotor; NOS, not otherwise specified; SMD, standardized mean difference.
